# Evaluation of RealStar® *Alpha* Herpesvirus PCR Kit for Detection of HSV-1, HSV-2, and VZV in Clinical Specimens

**DOI:** 10.1155/2019/5715180

**Published:** 2019-10-09

**Authors:** Cyril C. Y. Yip, Siddharth Sridhar, Kit-Hang Leung, Andrew K. W. Cheng, Kwok-Hung Chan, Jasper F. W. Chan, Vincent C. C. Cheng, Kwok-Yung Yuen

**Affiliations:** ^1^Department of Microbiology, Queen Mary Hospital, Hong Kong, China; ^2^Department of Microbiology, Li Ka Shing Faculty of Medicine, The University of Hong Kong, Hong Kong, China; ^3^State Key Laboratory of Emerging Infectious Diseases, The University of Hong Kong, Hong Kong, China; ^4^Research Centre of Infection and Immunology, The University of Hong Kong, Hong Kong, China; ^5^Carol Yu Centre for Infection, The University of Hong Kong, Hong Kong, China; ^6^Collaborative Innovation Center for Diagnosis and Treatment of Infectious Diseases, The University of Hong Kong, Hong Kong, China

## Abstract

Several commercial PCR kits are available for detection of herpes simplex virus (HSV) and varicella zoster virus (VZV), but the test performance of one CE-marked in vitro diagnostic kit—RealStar® *alpha* Herpesvirus PCR Kit—has not been well studied. This study evaluated the performance of RealStar® *alpha* Herpesvirus PCR Kit 1.0 on the LightCycler® 480 Instrument II for detection and differentiation of HSV-1, HSV-2, and VZV in human clinical specimens. We evaluated the analytical sensitivity of the RealStar® and in-house multiplex real-time PCR assays using serial dilutions of nucleic acids extracted from clinical specimens. The analytical sensitivity of the RealStar® assay was 10, 32, and 100 copies/reaction for HSV-1, HSV-2, and VZV, respectively, which was slightly higher than that of the in-house multiplex real-time PCR assay. Reproducibility of the cycle threshold (Cp) values for each viral target was satisfactory with the intra- and interassay coefficient of variation values below 5% for both assays. One-hundred and fifty-three clinical specimens and 15 proficiency testing samples were used to evaluate the diagnostic performance of RealStar® *alpha* Herpesvirus PCR Kit against the in-house multiplex real-time PCR assay. The RealStar® assay showed 100% sensitivity and specificity when compared to the in-house assay. Cp values of the RealStar® and in-house assays showed excellent correlation. RealStar® *alpha* Herpesvirus PCR is a sensitive, specific, and reliable assay for the detection of HSV-1, HSV-2, and VZV, with less extensive verification requirements compared to a laboratory developed assay.

## 1. Introduction

Herpes simplex virus- (HSV-) 1, HSV-2, and varicella zoster virus (VZV) are important pathogenic human herpesviruses. The manifestations of HSV-1, HSV-2, and VZV often overlap, making clinical differentiation difficult. For example, all three viruses can cause meningoencephalitis, keratoconjunctivitis, and retinitis. HSV-1 and HSV-2 both cause genital herpes, which can mimic perineal or sacral herpes zoster due to VZV [1]. Precise virological diagnosis is important as antiviral dosages and schedules for HSV-1, HSV-2, and VZV infections differ [2, 3]. Furthermore, confirming VZV infections has distinct infection control implications in healthcare settings as patients may require airborne isolation, especially in hematology or transplant wards.

Cell culture and direct fluorescence-antibody assay have been used for HSV and VZV detection, but they are less sensitive when compared to molecular tests [4–6]. Performing one-stop multiplex real-time HSV and VZV PCR assay is an attractive choice for molecular virology laboratories due to convenience, speed, user satisfaction, and lower manpower requirements [7–11]. In this study, we evaluated the performance of a commercially available RealStar® *alpha* Herpesvirus PCR Kit 1.0 capable of detecting and differentiating HSV-1, HSV-2, and VZV against our in-house developed multiplex PCR assay using archived clinical specimens and proficiency testing samples.

## 2. Materials and Methods

### 2.1. Samples Used for Evaluation

This study included 153 clinical specimens (Table 1) sent for HSV and/or VZV testing to the Microbiology Laboratory at Queen Mary Hospital in Hong Kong during March 2017 to July 2019. In addition to the clinical specimens used for assay validation, 15 samples with various concentrations of HSV/VZV or negative for HSV/VZV from College of American Pathologists (CAP) and Quality Control for Molecular Diagnostics (QCMD) were used for external quality assessment (EQA). This study was approved by the Institutional Review Board (IRB) of the University of Hong Kong/Hospital Authority Hong Kong West Cluster. The samples analyzed had been deidentified to staff undertaking the evaluation and no clinical or demographic details were analyzed. Hence, the need for informed consent from patients was waived by the IRB.

### 2.2. Viral Nucleic Acid Extraction

Samples were subjected to total nucleic acid (TNA) extraction by using the NucliSENS easyMAG extraction system (bioMérieux, France). The volume of the samples used for TNA extraction and the elution volume depended on the sample type and the amount of sample available (Table 2). Aqueous/vitreous biopsy was extracted by QIAamp DNA Mini Kit (QIAGEN, Germany), with the elution volume of 200 *μ*L, according to manufacturer's instructions.

### 2.3. RealStar® *Alpha* Herpesvirus PCR

The samples were run in parallel with the in-house multiplex real-time PCR using the RealStar® *alpha* Herpesvirus PCR Kit 1.0 (altona Diagnostics GmbH, Germany), according to the manufacturer's instructions. Briefly, PCR was performed using the kit reagents mixed with 5 *μ*L sample template and LightCycler® 480 Instrument II (Roche, Switzerland) with the following PCR conditions: 95°C for 10 min, followed by 45 cycles at 95°C for 15 s and 58°C for 1 min.

### 2.4. In-House Developed Multiplex Real-Time PCR

Primers and probes used for HSV-1, HSV-2, and VZV detection and monitoring PCR inhibition are shown in Table 3. A 20 *μ*L reaction mixture contained 5 *μ*L of 4x QuantiNova Multiplex PCR Master Mix (QIAGEN, Germany), 5 *μ*Lof nuclease-free water, 400 nM of each primer, 250 nM of each probe, 1 *μ*L of internal control plasmid DNA (1 × 10^3^ copies/*μ*L), and 5 *μ*L of sample template. The in-house multiplex real-time PCR assay was performed by LightCycler® 480 Instrument II (Roche, Switzerland). The PCR conditions consisted of 1 cycle at 95°C for 2 min, followed by 50 cycles at 95°C for 5 s and 60°C for 30 s. Plasmid controls were prepared using the pCRII-TOPO vector (Invitrogen, USA) cloned with target inserts (75 bp UL30 DNA polymerase gene of HSV-1, 117 bp envelope glycoprotein G gene of HSV-2 and 67 bp ORF29 DNA binding protein gene of VZV). Each plasmid stock was diluted in AE buffer, resulting in the concentrations of 1 × 10^4^ copies/reaction and 1 × 10^2^ copies/reaction as high and low positive controls, respectively. To monitor PCR inhibition in the in-house multiplex PCR assay, recombinant plasmid cloned with a 113 bp insert containing EGFP region flanked by primers derived from brome mosaic virus gene sequence of known concentration was diluted in AE buffer to be used as internal control (1 × 10^3^ copies/reaction) [12].

### 2.5. Analytical Sensitivity, Specificity, and Imprecision

Analytical sensitivity (limit of detection) was determined by evaluating a dilution series of TNA extracted from clinical specimens containing HSV-1 or HSV-2 or VZV. Ten-fold serial dilutions of each recombinant plasmid (HSV-1/HSV-2/VZV) were used to generate standard curves for measuring DNA concentration of original extracts of each virus. Each concentration was tested in 8 replicates for both RealStar® and in-house multiplex real-time PCR assays. Probit analysis was used to calculate the limit of detection (LOD) of the assays. Analytical specificity (cross reactivity) was determined by testing a panel of genomic DNA/RNA extracted from other herpesviruses or other pathogens significant in immunocompromised patients. Imprecision was evaluated by testing different concentrations of TNA extracted from clinical specimens containing each type of virus (in triplicate for each concentration in each run) in two independent runs.

## 3. Results

Performance characteristics of the RealStar® *alpha* Herpesvirus PCR Kit and the in-house multiplex real-time PCR assay for the detection of HSV-1, HSV-2, and VZV DNA were evaluated. LOD is defined as the concentration of viral DNA that can be detected with a positivity rate of 95% in this study. The LOD of the RealStar® assay was 10, 32, and 100 copies/reaction for HSV-1, HSV-2, and VZV, respectively, while that of the in-house multiplex real-time PCR assay was 24, 63, and 123 copies/reaction for HSV-1, HSV-2 and VZV, respectively (Table 4).

The analytical specificity of the RealStar® and in-house multiplex real-time PCR assays was evaluated. Both assays did not show cross reaction with cytomegalovirus, Epstein–Barr virus, human herpesvirus 6, 7, and 8, BK virus, JC virus, hepatitis B and C viruses, parvovirus B19, and human enterovirus. The HSV-1, HSV-2, and VZV primer/probe combinations within both multiplex PCR assays were target specific; both RealStar® and in-house assays are able to distinguish between HSV-1, HSV-2, and VZV.

In the replication experiment, three replicates of each concentration of TNA extracted from clinical specimens containing each viral target (HSV-1, HSV-2 or VZV) were tested to evaluate the intra- and interassay variations. The reproducibility of the Cp values for each target was satisfactory; total imprecision (% CV) values ranged from 0.80% to 1.94% for the RealStar® assay and 0.33% to 2.50% for the in-house multiplex PCR assay (Table 5).

**Figure 1:**
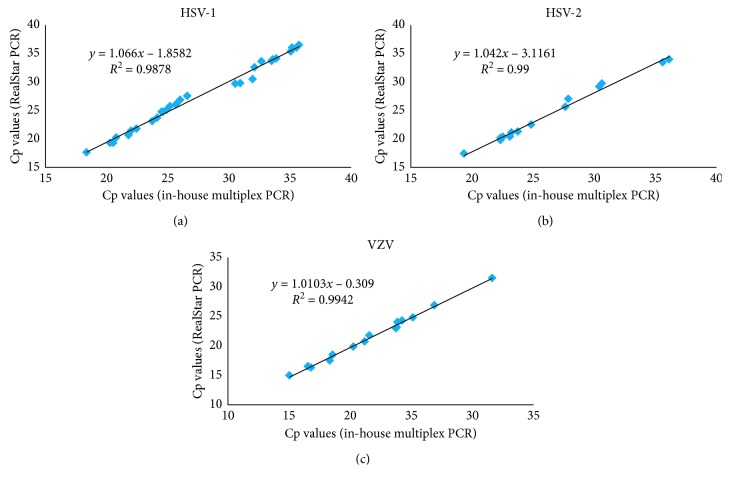
Correlation of the Cp values of the samples found positive for (a) HSV-1, (b) HSV-2, and (c) VZV by the RealStar® and in-house HSV and VZV multiplex real-time PCR assays.

Among the 153 clinical specimens and 15 samples for EQA subjected to HSV-1, HSV-2, and VZV detection, 60 were positive (31 HSV-1, 14 HSV-2, and 15 VZV) and 108 were negative by both RealStar® and in-house multiplex real-time PCR assays (Supplementary [Supplementary-material supplementary-material-1]). No PCR inhibition was observed in each reaction for both assays. Using the in-house multiplex real-time PCR assay as the reference method, the sensitivity and specificity of the RealStar® assay were 100% (Table 6). There was a good agreement in performance of the RealStar® assay compared to the in-house multiplex assay demonstrating strong correlation with a coefficient of determination (*R*^2^) of 0.99 for each target (Figure 1). For the EQA samples evaluation, both RealStar® and in-house multiplex real-time PCR assays could give 100% correct results for the proficiency testing samples from CAP and QCMD (Supplementary [Supplementary-material supplementary-material-1]).

## 4. Discussion

In recent years, several FDA-approved or CE-marked in vitro diagnostic (IVD) commercial PCR kits have been launched for the detection of HSV and VZV in clinical specimens, such as Focus Diagnostics (Cypress, CA), EraGen Biosciences, Inc. (Madison, WI), and Lyra Direct HSV 1 + 2/VZV assay (Quidel Corporation, CA), and their diagnostic performances have been well studied [13–15]. At the time of writing, another CE-IVD kit, RealStar® *alpha* Herpesvirus PCR Kit, has been used for resolving discordant results [11], but its performance characteristics have not been well evaluated. In this study, using a variety of specimens derived from patients and EQA samples, we were able to demonstrate that the diagnostic performance of the RealStar® *alpha* Herpesvirus PCR assay was equivalent to that of our in-house multiplex real-time PCR assay. Furthermore, we showed that the RealStar® *alpha* Herpesvirus PCR assay was noninferior to the in-house multiplex PCR assay in terms of analytical sensitivity. Reproducibility of the cycle threshold (Cp) values for each viral target was satisfactory with the intra-and interassay coefficient of variation values below 5% for these two assays. Cp values of both assays showed excellent correlation. The assays also performed well in proficiency testing samples from two EQA service providers. According to the package insert, the RealStar® *alpha* Herpesvirus PCR assay has been designed for use with several amplification instruments but not with LightCycler® 480 Instrument II. In the present study, the RealStar® assay was validated to be used with LC480 PCR system based on our evaluation results. Since LC480 PCR system provides 384-well format for high-throughput real-time PCR, it would be advantageous for reference laboratories that process large number of specimens every day. In a study on in-house quadruplex real-time PCR for rapid detection of human alpha herpesviruses, Krumbholz et al. have evaluated the matrix effect and effects of multiple infections [16], and these data can underline results on assay performance. Thus, studying these two effects is worthy to be included in the future studies, because this can make the evaluation of the assays more complete.

Compared to conventional PCR, real-time PCR minimizes the risk of amplicons carryover contamination, saves time, and enables estimation of viral load in clinical specimens. By combining multiple reaction targets in a single vessel, multiplex real-time PCR assays enable the laboratory to improve the turnaround time and optimize the manpower allocation [17]. Such multiplex testing facilitates diagnostic test panel-based reporting, which is advantageous when multiple pathogens can produce a similar clinical presentation. The RealStar® assay takes around 130 min; starting with the total nucleic acid extraction until the report of results, the time is a bit longer than the in-house assay which takes around 100 min. While commercial kits are easier to implement, developing tests in-house further enables the laboratory to significantly save on reagent costs. Our in-house multiplex real-time PCR assay costs US$ 2 per reaction compared to US$ 17.5 per reaction for the RealStar® *alpha* Herpesvirus PCR Kit.

The potential drawback of multiplex real-time PCR is the potential loss of sensitivity due to combination of multiple primers and probes (including internal control) in a single reaction. We did not observe this phenomenon with either the in-house multiplex real-time PCR or commercial kit when compared to in-house conventional monoplex nested PCR assay (data not shown). Another disadvantage is a loss in testing flexibility, i.e., laboratories are forced to test for VZV even when the clinical presentation is highly suggestive of HSV and vice versa. However, given the overlapping presentations and possibility of HSV/VZV coinfections, detection of both targets simultaneously may enable more precise diagnosis than selective PCR testing in some cases.

## 5. Conclusions

RealStar® *alpha* Herpesvirus PCR assay is highly sensitive, specific, and reliable for the detection and differentiation of HSV-1, HSV-2, and VZV in a variety of human clinical specimens.

## Figures and Tables

**Table 1:** Clinical specimens used for evaluation.

Specimen type	Number
Eye (anterior chamber aspirate, aqueous and vitreous tapping, swab, biopsy)	29
Genital swabs (penile, vaginal, vulval)	23
CSF	20
Mouth/oral swab, saliva	20
Plasma	20
Respiratory specimens	21
Others (blister fluid, lesion/skin/vesicle swabs, gastric fluid)	20

CSF, cerebrospinal fluid.

**Table 2:** Samples used for TNA extraction by the NucliSENS easyMAG extraction system.

Sample type	Sample volume (*μ*L)	Elution volume (*μ*L)
CSF, body fluids, aqueous/vitreous tapping, anterior chamber aspirate	200/100 or ≤50	50 or 30
Plasma	1000/500	25
^*∗*^Specimens in VTM	250	55

^*∗*^Genital swabs, oral swab, skin swab, vesicle fluid/swab, and respiratory specimens. VTM, viral transport medium.

**Table 3:** Primers and probes used for the in-house developed real-time multiplex PCR assay.

Target		Sequence (5′-3′)	Amplicon size
HSV-1	Forward primer	ATCGGCGAGTACTGCATACA	75 bp (UL30 DNA polymerase gene)
	Reverse primer	GAGCTCCAGATGGGGCAA	
	Probe	HEX-ATTCCCTGCTGGTGGGCCA-IABkFQ	

HSV-2	Forward primer	ACGCTCTCGTAAATGCTTCC	117 bp (envelope glycoprotein G gene)
	Reverse primer	CCACCTCTACCCACAACAGA	
	Probe	LC610-CGCGGAGACATTCGAGTACCAGATCG-BBQ	

VZV	Forward primer	CGAGAACGGTTTGGGTTT	67 bp (ORF29 DNA binding protein gene)
	Reverse primer	CGCCGGTCGTCTCAACAG	
	Probe	FAM-CGCTGCCAAGGGCCTCCTGTT-IABkFQ	

Internal control	Forward primer	GTTCACCGATAGACCGCTG	113 bp
	Reverse primer	AAGAGCCCGGAATGTCAAGA	
	Probe	Cy5-ACTACCTGAGCACCCAGTCCGCCCT-BBQ	

**Table 4:** PCR results used for calculation of analytical sensitivity.

Target	DNA concentration (copies/reaction)	Number of replicates	Number of positives	Hit rates (%)
RealStar® *alpha* Herpesvirus PCR assay				
HSV-1	6.04 × 10^1^	8	8	100
	3.02 × 10^1^	8	8	100
	1.51 × 10^1^	8	8	100
	3.78	8	5	62.5
	1	8	1	12.5
	No template control	8	0	0
HSV-2	1.76 × 10^2^	8	8	100
	8.80 × 10^1^	8	8	100
	4.40 × 10^1^	8	8	100
	1.10 × 10^1^	8	5	62.5
	2.75	8	2	25
	No template control	8	0	0
VZV	2.29 × 10^2^	8	8	100
	1.15 × 10^2^	8	8	100
	5.73 × 10^1^	8	6	75
	1.43 × 10^1^	8	2	25
	No template control	8	0	0

In-house multiplex PCR assay				
HSV-1	6.04 × 10^1^	8	8	100
	3.02 × 10^1^	8	8	100
	1.51 × 10^1^	8	7	87.5
	7.55	8	2	25
	3.78	8	2	25
	1	8	0	0
	No template control	8	0	0
HSV-2	1.76 × 10^2^	8	8	100
	8.80 × 10^1^	8	8	100
	4.40 × 10^1^	8	7	87.5
	2.20 × 10^1^	8	4	50
	1.10 × 10^1^	8	3	37.5
	2.75	8	0	0
	No template control	8	0	0
VZV	2.29 × 10^2^	8	8	100
	1.15 × 10^2^	8	7	100
	5.73 × 10^1^	8	6	75
	2.86 × 10^1^	8	3	37.5
	1.43 × 10^1^	8	0	0
	No template control	8	0	0

**Table 5:** Imprecision testing of the RealStar® and in-house multiplex real-time PCR assays using HSV-1, HSV-2, and VZV extracts.

DNA concentration (copies/reaction)		Intra-assay		Interassay
		No. of positive replicates	Mean Cp ± SD (% coefficient of variation)	Mean Cp ± SD (% coefficient of variation)
RealStar® *alpha* Herpesvirus PCR assay				
HSV-1				
6.04 × 10^2^		3	31.41 ± 0.12 (0.38)	31.38 ± 0.26 (0.84)
6.04 × 10^1^		3	33.99 ± 0.32 (0.93)	34.16 ± 0.36 (1.06)
HSV-2				
1.76 × 10^3^		3	30.90 ± 0.08 (0.26)	30.71 ± 0.26 (0.80)
1.76 × 10^2^		3	32.98 ± 0.08 (0.25)	33.37 ± 0.44 (1.30)
VZV				
2.29 × 10^3^		3	31.54 ± 0.13 (0.41)	31.84 ± 0.35 (1.11)
2.29 × 10^2^		3	34.42 ± 0.39 (1.12)	34.91 ± 0.68 (1.94)

In-house multiplex PCR				
HSV-1				
6.04 × 10^3^		3	30.79 ± 0.12 (0.39)	30.80 ± 0.10 (0.33)
6.04 × 10^2^		3	34.09 ± 0.15 (0.45)	34.09 ± 0.18 (0.52)
6.04 × 10^1^		3	37.46 ± 0.85 (2.28)	37.84 ± 0.85 (2.26)
HSV-2				
1.76 × 10^4^		3	30.57 ± 0.16 (0.52)	30.39 ± 0.23 (0.76)
1.76 × 10^3^		3	34.65 ± 0.21 (0.61)	34.20 ± 0.51 (1.50)
1.76 × 10^2^		3	37.79 ± 0.57 (1.52)	37.63 ± 0.56 (1.50)
VZV				
2.29 × 10^4^		3	30.64 ± 0.06 (0.18)	30.11 ± 0.58 (1.91)
2.29 × 10^3^		3	33.87 ± 0.36 (1.05)	33.14 ± 0.83 (2.50)
2.29 × 10^2^		3	36.09 ± 0.45 (1.25)	36.13 ± 0.40 (1.11)

**Table 6:** Diagnostic performance of the RealStar® assay compared to the in-house HSV and VZV multiplex real-time PCR assay.

	In-house multiplex real-time PCR assay				
	Positive	Negative	Total	Sensitivity % (95% CI)	Specificity % (95% CI)
RealStar*®* assay					
Positive	60	0	60	100 (94.0–100)	100 (96.6–100)
Negative	0	108	108		
Total	60	108	168		

## Data Availability

The data used to support the findings of this study are available from the corresponding author upon request.

## Supplementary Materials

Supplementary Materials Supplementary Table S1: the detection of HSV-1, HSV-2, and VZV by RealStar® and in-house multiplex real-time PCR assays using various types of clinical specimens.

## References

[1] Granato P. A., DeGilio M. A., Wilson E. M. (2016). The unexpected detection of varicella-zoster virus in genital specimens using the Lyra™ Direct HSV 1 + 2/VZV assay. *Journal of Clinical Virology*.

[2] Dworkin R. H., Johnson R. W., Breuer J. (2007). Recommendations for the management of herpes zoster. *Clinical Infectious Diseases*.

[3] Gnann J. W., Whitley R. J. (2016). Genital herpes. *New England Journal of Medicine*.

[4] Liu J., Yi Y., Chen W. (2015). Development and evaluation of the quantitative real-time PCR assay in detection and typing of herpes simplex virus in swab specimens from patients with genital herpes. *International Journal of Clinical and Experimental Medicine*.

[5] Madhavan H., Priya K., Anand A. R., Therese K. L. (1999). Detection of herpes simplex virus (HSV) genome using polymerase chain reaction (PCR) in clinical samples comparison of PCR with standard laboratory methods for the detection of HSV. *Journal of Clinical Virology*.

[6] Wilson D. A., Yen-Lieberman B., Schindler S., Asamoto K., Schold J. D., Procop G. W. (2012). Should varicella-zoster virus culture be eliminated? A comparison of direct immunofluorescence antigen detection, culture, and PCR, with a historical review. *Journal of Clinical Microbiology*.

[7] Bennett S., Carman W. F., Gunson R. N. (2013). The development of a multiplex real-time PCR for the detection of herpes simplex virus 1 and 2, varizella zoster virus, adenovirus and Chlamydia trachomatis from eye swabs. *Journal of Virological Methods*.

[8] Pillet S., Verhoeven P. O., Epercieux A., Bourlet T., Pozzetto B. (2015). Development and validation of a laboratory-developed multiplex real-time PCR assay on the BD max system for detection of herpes simplex virus and varicella-zoster virus DNA in various clinical specimens. *Journal of Clinical Microbiology*.

[9] Sankuntaw N., Sukprasert S., Engchanil C. (2011). Single tube multiplex real-time PCR for the rapid detection of herpesvirus infections of the central nervous system. *Molecular and Cellular Probes*.

[10] Tan T. Y., Zou H., Ong D. C. T. (2013). Development and clinical validation of a multiplex real-time PCR assay for herpes simplex and varicella zoster virus. *Diagnostic Molecular Pathology*.

[11] Wong A. A., Pabbaraju K., Wong S., Tellier R. (2016). Development of a multiplex real-time PCR for the simultaneous detection of herpes simplex and varicella zoster viruses in cerebrospinal fluid and lesion swab specimens. *Journal of Virological Methods*.

[12] Yip C. C. Y., Sridhar S., Cheng A. K. W. (2017). Comparative evaluation of a laboratory developed real-time PCR assay and the RealStar® HHV-6 PCR Kit for quantitative detection of human herpesvirus 6. *Journal of Virological Methods*.

[13] Buelow D. R., Bankowski M. J., Fofana D., Gu Z., Pounds S., Hayden R. T. (2013). Comparison of two multiplexed PCR assays for the detection of HSV-1, HSV-2, and VZV with extracted and unextracted cutaneous and mucosal specimens. *Journal of Clinical Virology*.

[14] Fan F., Stiles J., Mikhlina A., Lu X., Babady N. E., Tang Y.-W. (2014). Clinical validation of the Lyra direct HSV 1 + 2/VZV assay for simultaneous detection and differentiation of three herpesviruses in cutaneous and mucocutaneous lesions. *Journal of Clinical Microbiology*.

[15] Heaton P. R., Espy M. J., Binnicker M. J. (2015). Evaluation of 2 multiplex real-time PCR assays for the detection of HSV-1/2 and varicella zoster virus directly from clinical samples. *Diagnostic Microbiology and Infectious Disease*.

[16] Krumbholz A., Schäfer M., Lorentz T., Sauerbrei A. (2019). Quadruplex real-time PCR for rapid detection of human alphaherpesviruses. *Medical Microbiology and Immunology*.

[17] Schmutzhard J., Merete Riedel H., Zweygberg Wirgart B., Grillner L. (2004). Detection of herpes simplex virus type 1, herpes simplex virus type 2 and varicella-zoster virus in skin lesions. Comparison of real-time PCR, nested PCR and virus isolation. *Journal of Clinical Virology*.

