# Supplementary material for: Evaluation of RealStar® Alpha Herpesvirus PCR Kit for Detection of HSV-1, HSV-2, and VZV in Clinical Specimens
Source: Biomed Res Int. 2019 Oct 9;2019:5715180. doi: 10.1155/2019/5715180 (PMC6803750; doi:10.1155/2019/5715180)
Supplement: Supplementary Materials — Supplementary Table S1: the detection of HSV-1, HSV-2, and VZV by RealStar® and in-house multiplex real-time PCR assays using various types of clinical specimens. [file 5715180.f1.docx]

Supplementary Table S1: The detection of HSV-1, HSV-2 and VZV by RealStar® and in-house multiplex real-time PCR assays using various types of clinical specimens

| **Date** | **Sample no.** | **Specimen type** | **RealStar® PCR (Cp)** | **In-house multiplex real-time PCR (Cp)** |
| --- | --- | --- | --- | --- |
| 13 Mar 2017 | 418916 | CSF | - | - |
| 2 May 2017 | 432836 | Pleural fluid | HSV1 (27.55) | HSV1 (25.69) |
| 2 May 2017 | 432837 | Anterior lung fluid | HSV1 (26.04) | HSV1 (23.75) |
| 2 May 2017 | 432838 | Posterior gastric fluid | HSV1 (26.84) | HSV1 (24.64) |
| 16 May 2017 | 437919 | CSF | - | - |
| 17 Aug 2017 | 470503 | CSF | - | - |
| 1 Sep 2017 | M62 | CSF | - | - |
| 15 Sep 2017 | 082556 | CSF | - | - |
| 14 Oct 2017 | 483968 | CSF | - | - |
| 24 Nov 2017 | 495578 | CSF | - | - |
| 8 Dec 2017 | 800616 | Vesicle swab | VZV (31.55) | VZV (31.47) |
| 9 Dec 2017 | 110387 | CSF | - | - |
| 22 Dec 2017 | 804204 | Plasma | HSV1 (34.21) | HSV1 (33.89) |
| 7 Jan 2018 | 800524 | Genital ulcer swab | HSV2 (20.45) | HSV2 (22.57) |
| 10 Jan 2018 | 800875 | Left vitreous tapping | VZV (19.88) | VZV (20.12) |
| 12 Jan 2018 | 800991 | Penile swab | HSV2 (22.53) | HSV2 (24.89) |
| 12 Jan 2018 | 801030 | High vaginal swab | HSV2 (25.63) | HSV2 (27.67) |
| 16 Jan 2018 | 801313 | Genital swab | HSV2 (20.14) | HSV2 (22.38) |
| 19 Jan 2018 | 801583 | Vulval swab | HSV1 (20.71) | HSV1 (21.99) |
| 19 Jan 2018 | 801585 | BAL | HSV1 (29.80) | HSV1 (32.10) |
| 20 Jan 2018 | 801575 | Plasma | HSV1 (36.04) | HSV1 (35.54) |
| 26 Jan 2018 | 802138 | Vulval swab | HSV2 (21.08) | HSV2 (23.80) |
| 6 Feb 2018 | 802768 | Left facial swab | VZV (20.77) | VZV (21.02) |
| 10 Feb 2018 | 803156 | BAL | HSV1 (33.72) | HSV1 (33.61) |
| 19 Feb 2018 | 803613 | Vulval swab | HSV1 (17.64) | HSV1 (20.80) |
| 11 Mar 2018 | 413842 | NPA | - | - |
| 13 Mar 2018 | M198 | CSF | HSV1 (24.80) | HSV1 (24.52) |
| 15 Mar 2018 | M210 | CSF | HSV1 (25.75) | HSV1 (25.11) |
| 16 Mar 2018 | M211 | CSF | HSV1 (25.15) | HSV1 (24.91) |
| 20 Mar 2018 | M205 | CSF | HSV1 (32.57) | HSV1 (32.70) |
| 28 Mar 2018 | 806457 | Plasma | HSV1 (30.53) | HSV1 (30.52) |
| 11 Apr 2018 | 807699/700 | Left vitreous tapping | - | - |
| 16 Apr 2018 | 807986/87 | Anterior chamber aspirate | - | - |
| 16 Apr 2018 | 808287 | Skin swab | - | - |
| 17 Apr 2018 | 808000/01 | Right eye aqueous tapping | - | - |
| 17 Apr 2018 | 808014/15 | Sublingual ulcer swab | - | - |
| 17 Apr 2018 | 808052 | Prepuce swab | HSV2 (20.36) | HSV2 (23.25) |
| 18 Apr 2018 | 808176/77 | Left eye aqueous | - | - |
| 19 Apr 2018 | 808239/40 | Right vitreous biopsy | - | - |
| 20 Apr 2018 | 808285/86 | Left anterior chamber aspirate | - | - |
| 23 Apr 2018 | M241 | Eye swab | HSV1 (19.26) | HSV1 (20.29) |
| 23 Apr 2018 | M242 | Mouth swab | HSV1 (24.79) | HSV1 (25.21) |
| 23 Apr 2018 | M243 | Face swab | HSV1 (25.45) | HSV1 (25.99) |
| 23 Apr 2018 | 808388/89 | Left eye aqueous | - | - |
| 24 Apr 2018 | 808557/58 | Right eye vitreous | - | - |
| 24 Apr 2018 | 808560/61 | Right eye aqueous | - | - |
| 26 Apr 2018 | 808768/69 | Rt. Aqueous tapping | - | - |
| 26 Apr 2018 | 808772 | Oral swab | - | - |
| 28 Apr 2018 | 808911/13 | Right eye vitreous tapping | - | - |
| 28 Apr 2018 | 808915/17 | Right eye anterior chamber aspirate | - | - |
| 1 May 2018 | 808984/85 | Plasma | - | - |
| 7 May 2018 | 809400 | Plasma | - | - |
| 7 May 2018 | 809451/52 | Right eye aqueous humour | - | - |
| 8 May 2018 | 809563 | Right Chest blister fluid | - | - |
| 10 May 2018 | 809706/07 | Left eye aqueous fluid | - | - |
| 10 May 2018 | 809779/80 | Anterior chamber aspirate | - | - |
| 11 May 2018 | M276 | CSF | - | - |
| 14 May 2018 | 809993/94 | Anterior chamber aspirate | - | - |
| 16 May 2018 | 810124 | Vulval swab | HSV2 (29.17) | HSV2 (30.65) |
| 18 May 2018 | 810272 | Genital swab | HSV2 (19.78) | HSV2 (22.38) |
| 21 May 2018 | 810443 | Vesicle fluid | VZV (14.99) | VZV (14.86) |
| 21 May 2018 | 810451/52 | Skin swab (right thigh) | - | - |
| 21 May 2018 | 810468/69 | Anterior chamber aspirate | - | - |
| 23 May 2018 | 810551 | Genital ulcer swab | HSV2 (17.47) | HSV2 (19.39) |
| 23 May 2018 | 810605 | Vesicle swab | VZV (16.27) | VZV (16.61) |
| 24 May 2018 | 810607/08 | Left eye aqueous | - | - |
| 24 May 2018 | 810610/11 | Left eye cornea | - | - |
| 25 May 2018 | 810657/58 | Anterior chamber aqueous | - | - |
| 25 May 2018 | 810683/84 | Right eye aqueous tapping | - | - |
| 25 May 2018 | 810774 | Vulval swab | - | - |
| 26 May 2018 | 810718/19 | Plasma | - | - |
| 28 May 2018 | 811000 | CSF | VZV (26.91) | VZV (26.75) |
| 29 May 2018 | 810981 | Bronchial aspirate | - | - |
| 30 May 2018 | 811183/84 | Left eye vitreous tapping | - | - |
| 31 May 2018 | 811283/84 | Left eye aqueous tapping | - | - |
| 3 May 2018 | 811390 | BAL | HSV1 (36.09) | HSV1 (35.08) |
| 4 Jun 2018 | 811494/95 | BAL | HSV1 (23.77) | HSV1 (24.16) |
| 4 Jun 2018 | 811497 | Glans penis ulcer swab | HSV2 (33.99) | HSV2 (36.14) |
| 5 Jun 2018 | 811535/36 | ABD swab | VZV (24.83) | VZV (23.70) |
| 5 Jun 2018 | 811537 | Saliva | VZV (23.17) | VZV (23.67) |
| 5 Jun 2018 | 811552/762 | Plasma | VZV (22.97) | VZV (23.59) |
| 6 Jun 2018 | 811598 | Saliva | HSV1 (21.84) | HSV1 (21.96) |
| 6 Jun 2018 | 811599/600 | Skin swab | HSV1 (36.53) | HSV1 (35.76) |
| 6 Jun 2018 | 811648/49 | BAL | - | - |
| 13 Jun 2018 | 812083 | Saliva | - | - |
| 13 Jun 2018 | 812130 | BAL | - | - |
| 16 Jun 2018 | 812459 | Anterior chamber aspirate | - | - |
| 17 Jun 2018 | 812449 | Penile swab | HSV2 (33.45) | HSV2 (35.62) |
| 19 Jun 2018 | 812534 | Genital swab | HSV1 (25.75) | HSV1 (26.60) |
| 30 Jun 2018 | 813481 | BAL | HSV1 (29.74) | HSV1 (30.96) |
| 1 Jul 2018 | M324 | CSF | - | - |
| 2 Jul 2018 | 813539 | Lower lip lesion | - | - |
| 4 Jul 2018 | 813626 | Genital swab | - | - |
| 4 Jul 2018 | 813719/20 | Right eye aqueous | - | - |
| 5 Jul 2018 | 813792 | BAL | - | - |
| 6 Jul 2018 | 813860 | Genital swab | HSV1 (21.49) | HSV1 (22.46) |
| 9 Jul 2018 | 814014 | Penile swab | - | - |
| 10 Jul 2018 | 814013 | Vulval swab | HSV1 (20.22) | HSV1 (21.79) |
| 10 Jul 2018 | 814119/20 | BAL | - | - |
| 10 Jul 2018 | 814137/38 | Anterior chamber aspirate | - | - |
| 12 Jul 2018 | 814249/50 | Right eye vitreous tapping | VZV (16.50) | VZV (16.37) |
| 29 Jul 2018 | M342 | CSF | - | - |
| 24 Aug 2018 | 816954 | CSF | - | - |
| 19 Sep 2018 | 817733 | Plasma | - | - |
| 20 Sep 2018 | 817779 | Saliva | - | - |
| 8 Oct 2018 | 818423 | Oral swab | - | - |
| 11 Oct 2018 | 818552 | Plasma | - | - |
| 19 Oct 2018 | 818845 | Plasma | - | - |
| 31 Oct 2018 | 819289 | BAL | - | - |
| 7 Nov 2018 | 819598 | Skin swab | - | - |
| 9 Nov 2018 | 819648 | Plasma | - | - |
| 13 Nov 2018 | 819839 | Oral swab | - | - |
| 15 Nov 2018 | 819979 | CSF | - | - |
| 19 Nov 2018 | 820048 | Plasma | - | - |
| 20 Nov 2018 | 820167 | BAL | - | - |
| 21 Nov 2018 | 820180 | Plasma | - | - |
| 23 Nov 2018 | 820279 | Plasma | - | - |
| 26 Nov 2018 | 820383 | Skin swab | - | - |
| 26 Nov 2018 | 820427 | ETA | - | - |
| 27 Nov 2018 | 820488 | Plasma | - | - |
| 29 Nov 2018 | 820604 | Penile swab | - | - |
| 5 Dec 2018 | 820876 | Oral swab | - | - |
| 7 Dec 2018 | 820948 | BAL | - | - |
| 10 Dec 2018 | 820985 | Penile swab | - | - |
| 21 Dec 2018 | 821605 | Oral swab | - | - |
| 9 Jan 2019 | 800467 | Genital swab | - | - |
| 13 Feb 2019 | 802125 | Skin vesicle fluid | - | - |
| 11 Mar 2019 | 803202 | Oral swab | - | - |
| 14 Mar 2019 | 803348 | Oral swab | - | - |
| 14 Mar 2019 | 803363 | CSF | - | - |
| 14 Mar 2019 | 803391 | Oral swab | - | - |
| 16 Mar 2019 | 803442 | Genital swab | - | - |
| 18 Mar 2019 | 803461 | Oral swab | - | - |
| 25 Mar 2019 | 803847 | Oral swab | - | - |
| 27 Mar 2019 | 803960 | Genital swab | - | - |
| 28 Mar 2019 | 804021 | Oral swab | - | - |
| 2 Apr 2019 | 804283 | Vesicle trunk swab | - | - |
| 16 Apr 2019 | 804897 | Plasma | - | - |
| 16 Apr 2019 | 804950 | Blister fluid | - | - |
| 17 Apr 2019 | 804978 | Plasma | - | - |
| 23 Apr 2019 | 805162 | BAL | - | - |
| 25 Apr 2019 | 805354 | Plasma | - | - |
| 26 Apr 2019 | 805363 | Plasma | - | - |
| 26 Apr 2019 | 805391 | BAL | - | - |
| 26 Apr 2019 | M522 | CSF | - | - |
| 29 Apr 2019 | 805499 | BAL | - | - |
| 30 Apr 2019 | 805562 | BAL | - | - |
| 30 Apr 2019 | 805575 | Blister swab | - | - |
| 18 Jun 2019 | 807837 | Plasma | - | - |
| 18 Jun 2019 | 807897 | Vesicle fluid | - | - |
| 20 Jun 2019 | 808041 | Saliva | - | - |
| 27 Jun 2019 | 808413 | Blister swab | - | - |
| 2 Jul 2019 | 808608 | Oral swab | - | - |
|  | CAP2016 ID1-01 |  | VZV (24.04) | VZV (24.97) |
|  | CAP2016 ID1-05 |  | HSV1 (23.12) | HSV1 (25.14) |
|  | CAP2016 ID1-09 |  | VZV (17.48) | VZV (18.19) |
|  | CAP2016 ID1-13 |  | HSV2 (21.32) | HSV2 (23.14) |
|  | CAP2017 ID1-01 |  | VZV (18.48) | VZV (18.39) |
|  | CAP2017 ID1-05 |  | HSV1 (19.29) | HSV1 (20.54) |
|  | CAP2017 ID1-09 |  | VZV (24.28) | VZV (24.09) |
|  | CAP2017 ID1-13 |  | - | - |
|  | CAP2018 ID1-01 |  | VZV (21.79) | VZV (21.39) |
|  | CAP2018 ID1-05 |  | HSV1 (21.23) | HSV1 (18.38) |
|  | HSVDNA18C1-01* | Transport medium | HSV1 (35.38) | HSV1 (35.18) |
|  | HSVDNA18C1-02* | Transport medium | HSV2 (29.76) | HSV2 (30.45) |
|  | HSVDNA18C1-03* | Transport medium | HSV1 (33.61) | HSV1 (33.53) |
|  | HSVDNA18C1-04* | Transport medium | HSV2 (27.02) | HSV2 (27.91) |
|  | HSVDNA18C1-05* | Transport medium | HSV1 (34.02) | HSV1 (31.95) |

BA, bronchial aspirate; BAL, bronchoalveolar lavage; CSF, cerebrospinal fluid; ETA, endotracheal aspirate; NPA, nasopharyngeal aspirate

+: detected; −: not detected

* samples from QCMD in 2018
